# Supplementary figures and images for: Adjunct Therapy with T Regulatory Cells Decreases Inflammation and Preserves the Anti-Tumor Activity of CAR T Cells
Source: Cells. 2023 Jul 18;12(14):1880. doi: 10.3390/cells12141880 (PMC10377823; doi:10.3390/cells12141880)

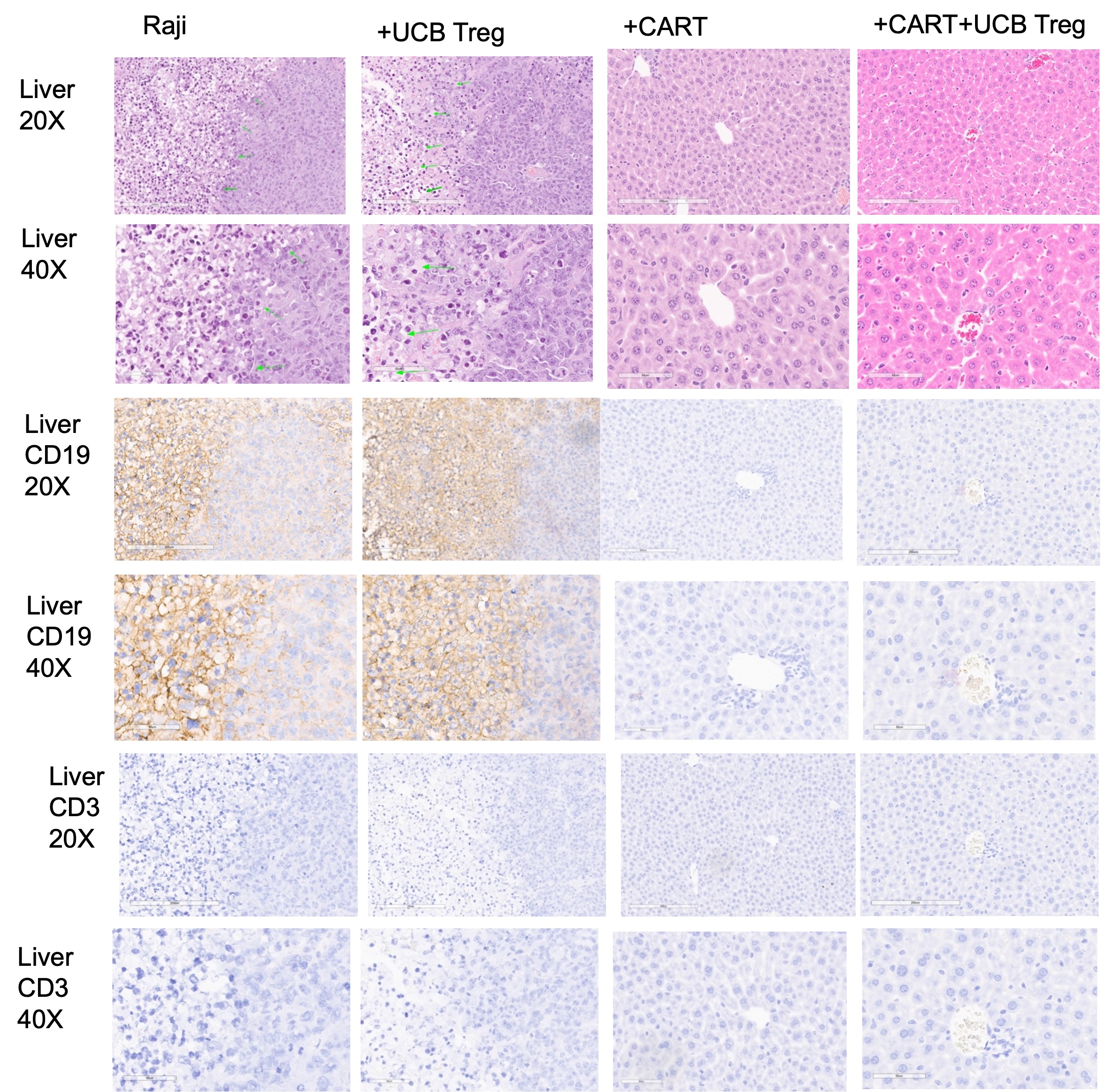

Supplement: Supplementary file 1 [file cells-12-01880-s001.zip › Figure S1.jpg]

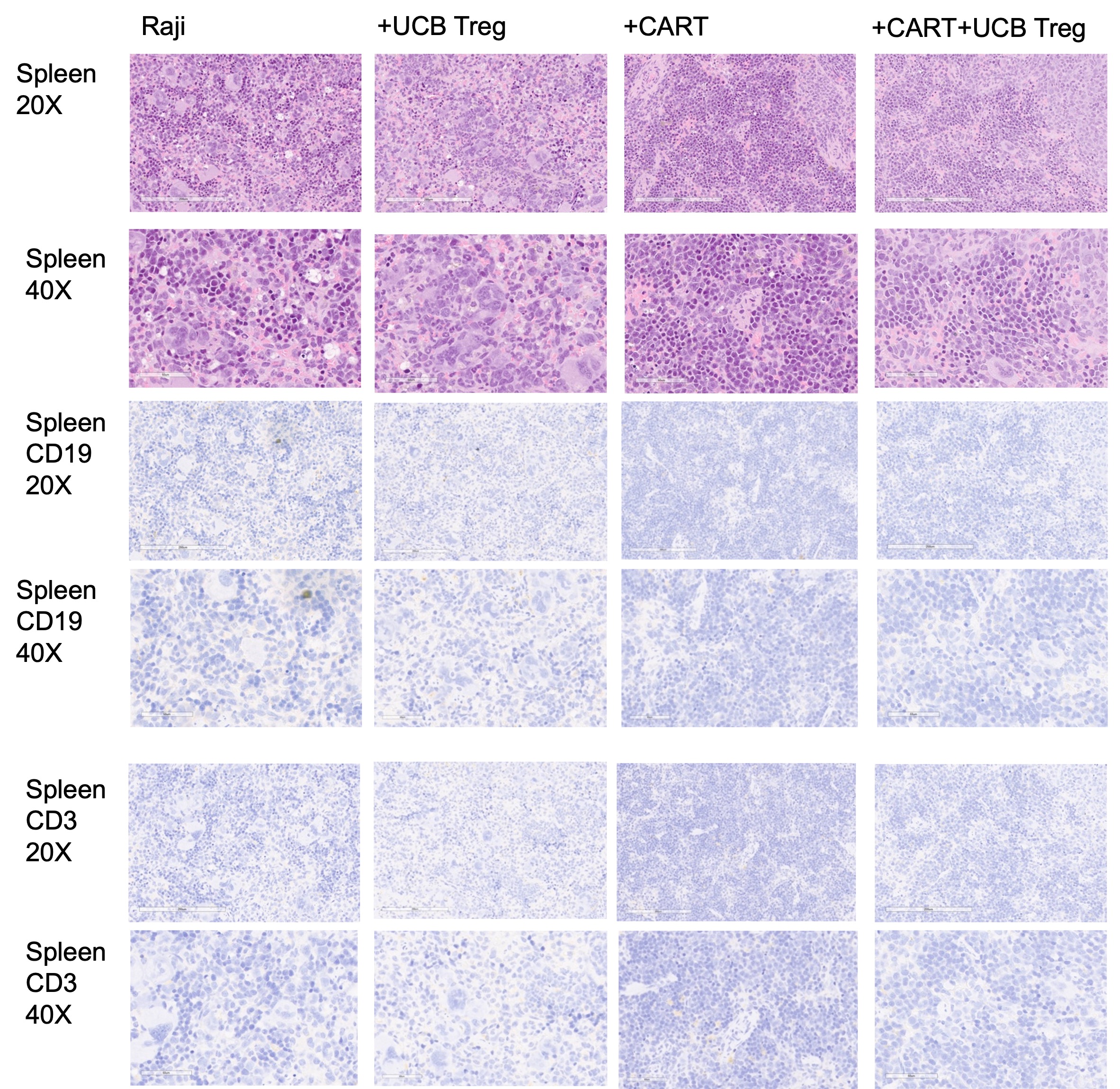

Supplement: Supplementary file 1 [file cells-12-01880-s001.zip › Figure S2.jpg]

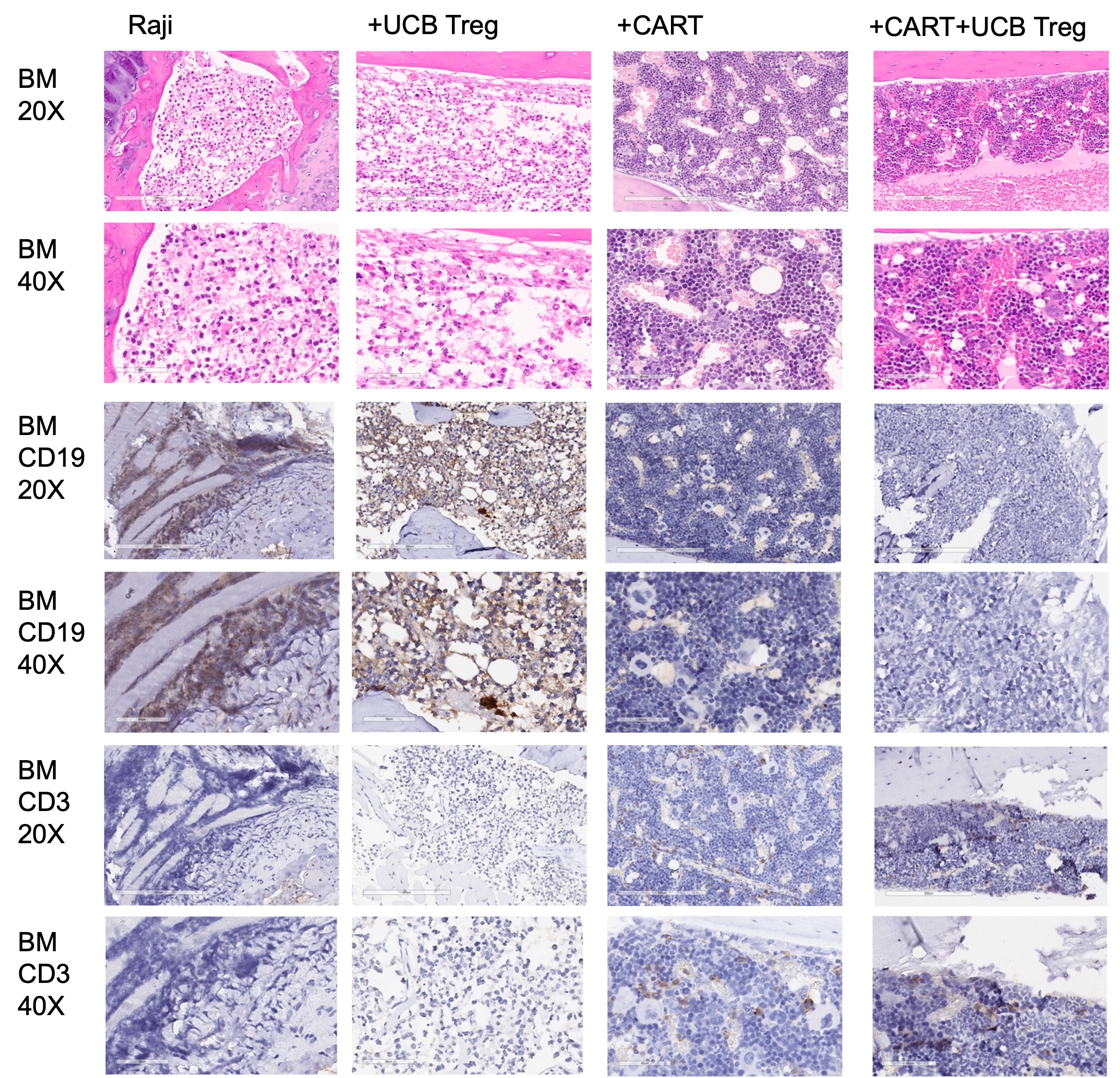

Supplement: Supplementary file 1 [file cells-12-01880-s001.zip › Figure S3.jpg]

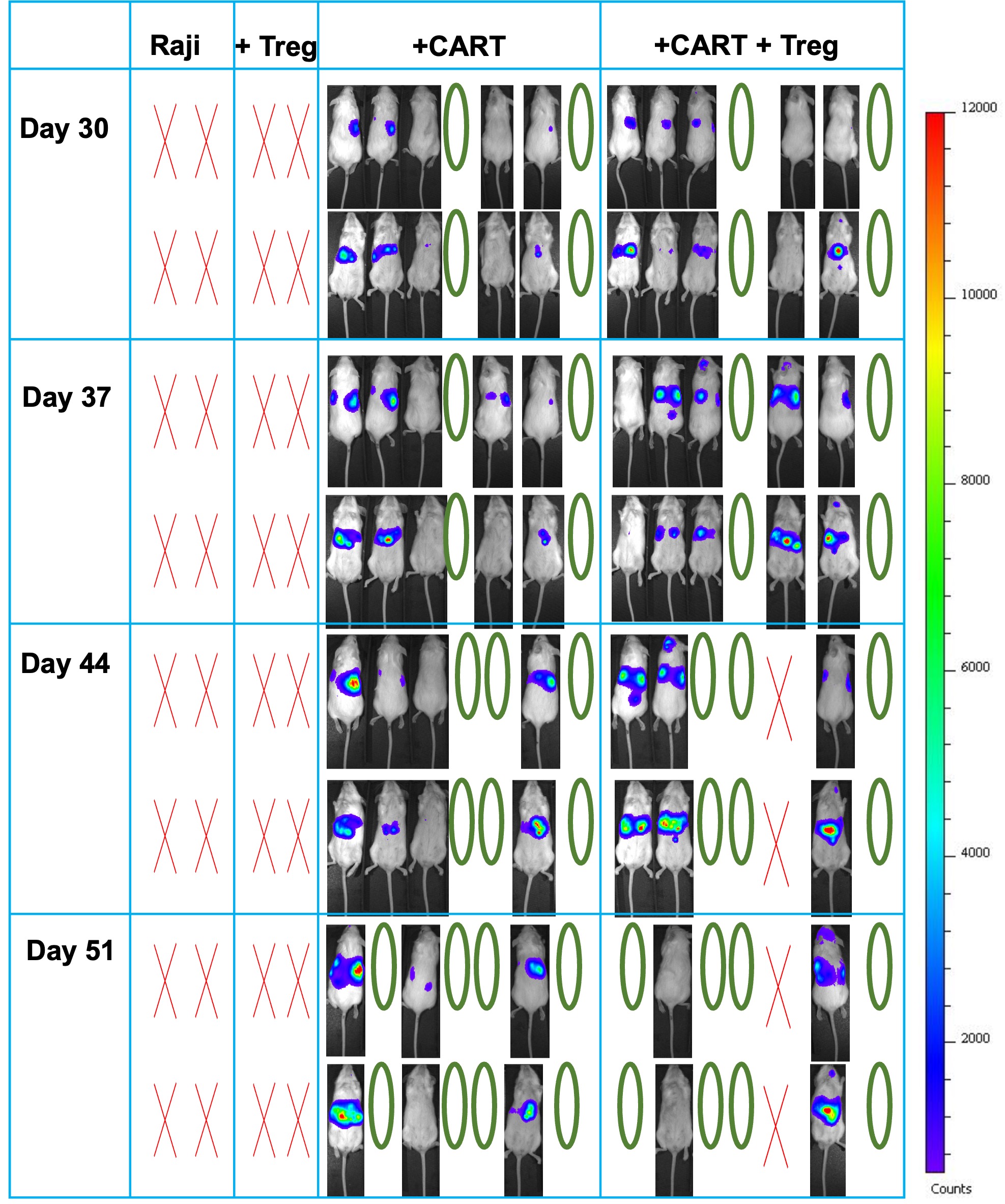

Supplement: Supplementary file 1 [file cells-12-01880-s001.zip › Figure S4.jpg]
